# Aging and Cardiovascular Risk

**DOI:** 10.1155/2015/871656

**Published:** 2015-03-02

**Authors:** Elísio Costa, Alice Santos-Silva, Constança Paúl, Javier González Gallego

**Affiliations:** ^1^Laboratory of Biochemistry, Department of Biological Sciences, Faculty of Pharmacy, University of Porto, Rua Jorge Viterbo Ferreira 228, 4050-313 Porto, Portugal; ^2^Institute of Molecular and Cell Biology (IBMC), University of Porto, Rua do Campo Alegre 823, 4150-180 Porto, Portugal; ^3^Abel Salazar Biomedical Sciences Institute, University of Porto, Rua Jorge Viterbo Ferreira 288, 4050-313 Porto, Portugal; ^4^UNIFAI, Unit of Research and Training in Adults and Elderly, University of Porto, Rua Jorge Viterbo Ferreira 228, 4050-313 Porto, Portugal; ^5^Institute of Biomedicine (IBIOMED), University of León, Campus Universitario, 24071 León, Spain

As part of the aging process, our bodies accumulate increasing damage at the tissue, cellular and molecular levels, leading to a loss of function and to an increased risk of morbidities and of mortality. Increasing life expectancy has been associated with increasing risk of aging-associated diseases, including cardiovascular disease (CVD). CVD is responsible for over than 4 million deaths in Europe every year.

The majority of CVD is caused by risk factors that can be controlled, treated, or modified, such as high blood pressure, cholesterol, overweight/obesity, tobacco use, lack of physical activity, and diabetes. However, there are also other major CVD risk factors that cannot be controlled. The normal process of aging is associated with progressive deterioration in structure and function of the heart and vasculature that likely contribute to the development of CVD, including coronary heart disease, hypertension, and heart failure. Since elderly is one of the fastest growing segments of the population, it is of vital importance that we have a thorough understanding of the physiological changes that occur with aging that could contribute to the increased risk for CVD in elderly population. More investigation at both the basic and clinical levels is necessary to identify therapies that will benefit older patients on the basis of both the pathophysiology of age-related CVD and the frequent presence of comorbid diseases.

This special issue includes review and original research articles. In one article, E. Bronze-da-Rocha reviews the biogenesis and processing of miRNAs, as well as their release, stability, and modulation. The potential use of miRNAs expression profiles as biomarkers for some human heart diseases and aging is highlighted. In another review article, G. D. Kolovou et al. reviewed some of mechanisms associated with ageing, such as the pathways involved in oxidative stress, lipid and glucose metabolism, inflammation, DNA damage and repair, growth hormone axis, and insulin-like growth factor, and some environmental factors. Moreover, some theories of ageing were also discussed. J. Wu et al. reviewed the role of oxidative stress and inflammation in cardiovascular aging. M. Janić et al. reviewed the different pharmacological therapeutic options for decreasing arterial stiffness. The influence of several groups of drugs is described: antihypertensive drugs, statins, peroral antidiabetics, advanced glycation end-products cross-link breakers, anti-inflammatory drugs, endothelin-A receptor antagonists, and vasopeptidase inhibitors. Concerning the research articles, Y. Chen et al. report an* in vitro* study showing that icariin, an important and active component in* Herba Epimedii*, upregulated the expression of* SIRT6* gene and had an inhibitory effect on NF-*κ*B inflammatory signaling pathways. These results provided some evidence that icariin could reduce the body's inflammatory response and delay aging. S. Coimbra et al. showed that adiponectin and leptin levels in elderly patients with type 2 diabetes mellitus are closely linked to obesity and to the length of the disease. Moreover, they showed that circulating chemerin concentrations are increased and are independent of the length of the disease and of the body mass index, suggesting that adipocyte dysfunction is enhanced with aging. M. d. Sameiro-Faria et al. showed that in the end-stage renal disease patients under hemodialysis bilirubin seem to confer protection for cardiovascular diseases, independently of age. C. Rammos et al. showed that nitrate dietary supplementation is associated with a reduction of circulating proinflammatory cytokine macrophage migration inhibitory factor levels, which is associated with an improvement in vascular function. This work supports the concept of dietary approaches to modulate age-related changes of vascular functions. C.-J. Lee et al. evaluate the relationship between fasting osteopontin serum concentration and carotid-femoral pulse wave velocity in geriatric persons. They showed that osteopontin, which seems to have a role in atherosclerosis, was an independent predictor of carotid-femoral pulse wave velocity in geriatric persons. J. M. Morillas-Ruiz et al. showed that the use of polyunsaturated fat at breakfast is advisable in women at risk of CVD, since margarine improved the plasma lipid profile. A. Gawron-Skarbek et al. reported no differences in total antioxidant activity between men with and without coronary heart disease. They also demonstrated no changes of the antioxidant capacity of human blood serum with age. M. A. Meraz-Ríos et al. demonstrated that allele 18-vWA (von Willebrand factor) and 9-human thyroid peroxidase (TPOX) and 12-TPOX are related with high venous thromboembolism risk, by using a case-control study. C. E. Wyers et al. showed that with increasing age the prevalence of CVD, venous thromboembolic events, hypertension, and diabetes mellitus type 2 increased up to half in men older than 70 years and in women older than 80 years. M. E. Mortby et al. provide further evidence of the protective effect of education for brain health and cognition. They also highlighted the importance of considering the possible interactive effects of comorbid CVD risk factors in increasing the risk of white matter pathology, irrespective of the protective effect provided by education-related cognitive and neural reserve. Finally, Y. H. Kim et al. describe the possibility of using Hachinski ischemic score in the community dwelling elderly population for the evaluation of the quantity of vascular factors. Association of high Hachinski ischemic score in, elderly community dwelling, with lower cognitive function, especially in elderly with poorly controlled vascular factors, was also demonstrated.

The present issue constitutes an important update in a constantly developing field. We hope this special issue will provide new inputs for those who are interested in aging and/or in cardiovascular risk factors.

*Elísio Costa* *Alice Santos-Silva* *Constança Paúl* *Javier González  Gallego*

